# Sandwich Multi-Material 3D-Printed Polymers: Influence of Aging on the Impact and Flexure Resistances

**DOI:** 10.3390/polym13224030

**Published:** 2021-11-21

**Authors:** Ana C. Pinho, Ana P. Piedade

**Affiliations:** Department of Mechanical Engineering, CEMMPRE, University of Coimbra, 3030-788 Coimbra, Portugal; acdspinho@uc.pt

**Keywords:** additive manufacturing, multi-material, sandwich structures, artificial saliva, mechanical properties, oral devices

## Abstract

With the advances in new materials, equipment, and processes, additive manufacturing (AM) has gained increased importance for producing the final parts that are used in several industrial areas, such as automotive, aeronautics, and health. The constant development of 3D-printing equipment allows for printing multi-material systems as sandwich specimens using, for example, double-nozzle configurations. The present study aimed to compare the mechanical behavior of multi-material specimens that were produced using a double-nozzle 3D printer. The materials that were included in this study were the copolymer acrylonitrile-butadiene-styrene (ABS), high-impact polystyrene (HIPS), poly(methyl methacrylate) (PMMA), and thermoplastic polyurethane (TPU). The configuration of the sandwich structures consisted of a core of TPU and the outer skins made of one of the other three materials. The mechanical behavior was evaluated through three-point bending (3PB) and transverse impact tests and compared with mono-material printed specimens. The effect of aging in artificial saliva was evaluated for all the processed materials. The main conclusion of this study was that the aging process did not significantly alter the mechanical properties for mono-materials, except for PMMA, where the maximum flexural stress decreased. In the sandwich structures, the TPU core had a softening effect, inducing a significant increase in the resilience and resistance to transverse impact. The obtained results are quite promising for applications in biomedical devices, such as protective mouthguards or teeth aligners. In these specific applications, the changes in the mechanical properties with time and with the contact of saliva assume particular importance.

## 1. Introduction

Over the last 50 years, fast and continuous progress in the manufacturing industry has been achieved due to advances in manufacturing processes [1]. These manufacturing processes can be divided into two major groups: subtractive (SM) and additive manufacturing (AM). The first operates by removing the excess bulk material using the top-down approach. On the other hand, AM is a term that defines all the manufacturing processes in which material is added successively, by layers, to create three-dimensional parts and, therefore, with a more sustainable bottom-up methodology [2]. AM also stands out compared to other modern manufacturing techniques because of the wide range of materials that can be used, such as plastics, ceramics, metal alloys, paper, and sand [3]. During fabrication with any AM process, the waste material was found to be minimal compared with SM. Consequently, AM techniques became very attractive from an environmental perspective due to the reduced amount of raw material needed to produce a part/component due to having less waste being produced [4]. Moreover, due to the possibility of printing dissimilar materials, the need for chemical modification with hazardous procedures is avoided [5,6]. Nowadays, AM can impact the manufacturing world, with it being a core element of Industry 4.0 [7].

The production of parts via AM begins with creating a digital model of the object using CAD software, followed by a slicing program [8]. The development of software that allows for generative design and topology optimization to create more lightweight and organic-shaped components with the same mechanical properties has increased the consolidation of AM processes for final parts production and will go further than prototype development. 

Among the different AM processes, the most commonly known is fused filament fabrication (FFF) in which a continuous filament of a thermoplastic polymer or polymer-based composite is used to successively print layers of materials on top of each other to build the final piece [9]. FFF benefits from being a relatively low-cost, high-speed, and straightforward process. However, FFF has some drawbacks, such as anisotropic mechanical properties and sometimes poor surface quality [10]. One of the solutions that was envisaged for enhancing the mechanical properties of 3D-printed parts was achieved via the development of fiber-reinforced composites using FFF [11]. However, this solution also introduces some problems, such as fiber orientation, bonding between the fiber and matrix, and void formation, as challenges when producing the 3D-printed parts [12].

Therefore, in recent years, the FFF of multi-material polymers has caught the attention of both the industry and academic community as a possible solution to overcome the stated problems [13]. With multiple materials, it is possible to enhance the mechanical properties, enable new functionalities for the printed parts and components, or even combine polymers with very distinct characteristics and properties [14]. One example is the simultaneous printing of polymers with poly(vinyl alcohol) (PVA). PVA is a water-soluble material with lower mechanical properties that can be used to create supports during printing a part [15]. When the 3D object has been created, the support material can be easily removed, adding more geometric complexity to the final part [16].

Recently, several works described finite element analysis (FEA) or artificial intelligence (AI) to predict the properties of printed structures with computational models. In the literature, the uses of these models are mainly via two approaches. In the first one, printed parts are modeled as solid parts with some homogenized effective properties. This means that the rasters are not modeled explicitly [17]. Other works include those where the microstructure was modeled explicitly, with the similarity of the structure obtained in FFF as high as possible [18,19]. Like the experimental and analytical approaches, FEA presents disadvantages, mainly because they compromise the understanding of the structure–property relationship and hide deformation mechanisms [17,18,19,20,21,22,23]. Nevertheless, some described works explicitly capture the anisotropy in printed parts by assigning orthotropic material properties to each raster [24,25]. Most of the published works are limited to mono-materials, particularly poly(lactic acid) (PLA) or PLA-based composite filaments.

In a different approach, and considering only the experimental characterization, multi-material 3D printing can also be used for the fabrication of sandwich structures. Sandwich structures include a core material and outer shells (skins) that can be made from the same or a different material to the core.

The performance of the final parts also depends on the properties of the skins and core, the adhesion between them, and the geometry of the final part [26]. The deposition parameters also influence the mechanical properties of the final part. For example, to maximize Young’s modulus and stiffness, the printed layers should be oriented along the loading line, and in order to promote the best performance in strength, stiffness, and ductility, the building orientation should be on-edge [27,28]. Lopez et al. [13] evaluated the tensile properties of different material combinations of sandwich structures that were produced using FFF. They tested poly(lactic acid) (PLA), ABS, and HIPS in different combinations. The results showed that the combination of an ABS core with PLA skins presented the best results, demonstrating the capability of FFF to produce one part with enhanced mechanical properties using two different materials. Singh et al. [29] evaluated the tensile properties of specimens that were composed of different PLA, HIPS, and ABS combinations. They observed that, while HIPS had the lowest strength of all the tested materials, when printed as a multi-material specimen with equal parts of PLA and ABS, there was an improvement in the tensile strength (from 27.4 to 28.8 kg/m^2^).

Brischetto et al. [30] studied the mechanical behavior of polymeric sandwich specimens with honeycomb cores. For this study, specimens were fully printed with a single material with different printing patterns for distinct parts of the specimen and a multi-material printer with distinct materials for the core and skins. Specimens were printed with PLA and ABS. The skins were printed using a linear pattern, while the core of the specimen was printed either with a honeycomb or a linear infill geometry. When utilizing two different extruders to print a PLA core with hexagonal infill and ABS skins, lower values for the elastic modulus were attained. The researcher attributed the result to the poor adhesion between the skins and the core. This effect was minimized using a 100% linear infill PLA core to promote higher adhesion between the different materials.

The present work aimed to create sandwich structures via multi-material 3D printing using the soft TPU as the core material and ABS, PMMA, and HIPS as skins. The bending and impact resistances were assessed for the mechanical properties using three-point bending tests and transverse impact tests, respectively. The materials were tested first in the mono-material configuration and subsequently with a multi-material configuration. Furthermore, the effect of an aging process using artificial saliva was explored, given the possibility of using such structures as medical protective devices, such as protective mouthguards, which are currently made with ethylene vinyl acetate (EVA) using conventional techniques [31].

## 2. Materials and Methods

### 2.1. Materials

The polymeric filaments that were used in the present work were commercially purchased and used without further modifications: DoWire^®^, Seixal, Portugal, provided the ABS and HIPS; TPU and PMMA were acquired from TreeD Filaments™, Seregno, Italy. All filaments had a diameter of 1.75 mm. 

### 2.2. Processing Using 3D Printing

All the printed specimens were produced using a FlashForge^TM^ Creator 3 3D printer with a dual extruder, each with a 0.4 mm diameter nozzle. A layer height of 0.18 mm was kept constant for all the printed specimens. For both mono- and multi-material printed parts, the 100% line (45°/−45°) infill pattern was used in order to obtain a better tension distribution and less anisotropic mechanical behavior [32].

**Figure 1:**
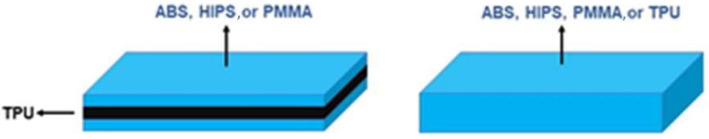
Schematic representation of the printed sandwich structures with ABS, HIPS, or PMMA as skins, and TPU as the core material (**left**) and mono-material specimens (**right**).

The choice of the infill pattern was intended to ensure the full coverage of each printed layer. In the sandwich structures, the core section was always TPU, while the skins were ABS, HIPS, or PMMA (Table 1). Although for structural applications, the core material is usually stiffer than the outer layers, herein, the choice of the material according to the sandwich configuration was based on the scope of the present work, where it was intended that the outer layers were more mechanically resistant, while the core should be able to better dissipate impact energy. The upper and lower skins were made of the same material, and each section represented one-third of the total height of the specimen, as represented in Figure 1. 

Despite the printing speed optimization tests that were performed for mono-material specimens, for sandwich structures, the slowest printing speed among the filaments was chosen in order to avoid the creation of extra tension at the interface between the core and outer shells. For each type of configuration and mechanical test, 12 samples were printed.

### 2.3. Aging Process

After printing, half (six) of the printed mono- and multi-material samples underwent an aging process in artificial saliva (AS) prior to the mechanical testing. For the aging process, an AS solution was prepared by dissolving in 800 mL of distilled water 0.426 g of disodium hydrogen phosphate (Na_2_HPO_4_), 1.68 g of sodium bicarbonate (NaHCO_3_), 0.147 g of calcium chloride (CaCl_2_), and 2.5 mL of hydrochloric acid (1M). Prior to the mechanical testing, the samples were individually placed in Falcon^®^ graduated tubes with 12 mL of the prepared AS solution and left inside a ThermoShaker (Incubator Shaker THO 500/1 from Gerhardt) for 14 days at a constant temperature of 37 °C and 100 rotations per minute (rpm). The 14 days are equivalent to the number of hours of device use considering 1 h per day over one year (336 h), while the 37 °C corresponds to the average human body temperature.

### 2.4. Mechanical Characterization of the Printed Specimens

#### 2.4.1. Three-Point Bending Tests

The bending resistances of the aged and non-aged printed specimens were assessed using three-point bending (3PB) tests according to the standard ASTM D790. Twelve 60 × 10 × 2 mm specimens of each mono- and multi-material configuration were printed. All tests were conducted using a Shimadzu AG-10 universal testing machine with a 5 kN load cell under a displacement rate of 2 mm·min^−1^ at room temperature with a span of 40 mm. The data collection was performed using Trapezium X software. The bending strength was determined as the nominal stress in the middle of the span section of the specimen, which was obtained using the load maximum value. The nominal bending was calculated according to Equation (1):

(1) $$\sigma =\frac{3PL}{2bh^{2}}$$

where *P* is the load; *L* is the span length; and *b* and *h* are the width and thickness of the specimen, respectively [33]. The calculation of Young’s modulus was performed according to the linear bending beams theory relationship (Equation (2)):

(2) $$E=\frac{∆PL^{3}}{48∆\mu I}$$

where ∆P and ∆μ are the load and the flexural displacement range in the middle of the span, respectively, for the interval of the linear part of the load-displacement plot and *I* is the inertia moment of the cross-section [34]. The ∆P/∆μ values were determined through the linear regression of the load versus displacement curves within the range of the linear segment considering a correlation factor greater than 95%. The obtained results are shown as mean ± standard deviation values.

#### 2.4.2. Transverse Impact Tests

The transverse impact test specimens were printed with the dimensions recommended by the standard Charpy ISO 179, i.e., 80 × 10 × 2 mm with a 2 mm “V” shaped notch. Twelve specimens of each mono- and multi-material conformation were considered, with half of them being previously aged, as aforementioned. The tests were carried out in an Instron Ceast 9050 impact machine that was equipped with a 5 J hammer at room temperature. The morphology of the damage was evaluated using a Carl ZEISS model Stemi 2000-c microscope, with a magnification factor of 5 times. A digital camera from CANON, model POWERSHOT G5, with a magnification factor of up to 16 times was used for the macrograph acquisition.

## 3. Results and Discussion

In the present work, all filaments were used as received from the suppliers. Fourier-transformed infrared spectroscopy (FTIR) was used to validate the chemical composition of the materials. The results confirmed that all filaments presented the expected functional chemical groups according to their chemical composition. Furthermore, thermal characterization using differential scanning calorimetry (DSC) confirmed that all filaments presented an amorphous structure. The printing temperatures were chosen according to the recommendations of the suppliers.

### 3.1. Saliva Induced Aging

Previously to the study of the influence of the aging process on the mechanical behavior of the printed specimens, dimensional evaluation was performed to detect possible volume and geometry changes that may have been induced by the AS environment. Both mono- and sandwich-printed specimens were measured before and after the aging process. The two types of specimens for mechanical testing were considered. The dimension data is shown in Table 2.

The obtained results showed no significant variation in the dimensions due to the immersion in artificial saliva, which confirmed that thermoplastic polymers used in this work show low affinity with aqueous solutions, which agrees with the literature data [35,36,37,38]. The ability to maintain the original dimensions after an aging process may be a critical factor in applications where dimensional variations can compromise the performance of the device, such as protective mouthguards or teeth aligners. Moreover, there was no dimension mismatch between the materials of the sandwich structures that could jeopardize the adhesion of the layers to each other. The chemical composition of the aged filaments was assessed using FTIR. No significant alterations were observed in the aged spectra.

### 3.2. Mechanical Properties of Printed Mono-Materials

Exposure to body solutions must be considered in several applications, which often brings new challenges in designing and conceptualizing components and the choice of materials. Herein, the maintenance of the mechanical properties during or after the contact with water or aqueous solutions is crucial for selecting the materials to use [39,40]. To simulate these conditions, the printed materials were submitted to an aging process that consisted of immersion in an artificial saliva solution at 37 °C. Both the aged and non-aged specimens were tested and their mechanical properties were calculated. For better understanding, the discussion of the results from mechanical characterization considers the mono-material and sandwich structures separately.

**Figure 2:**
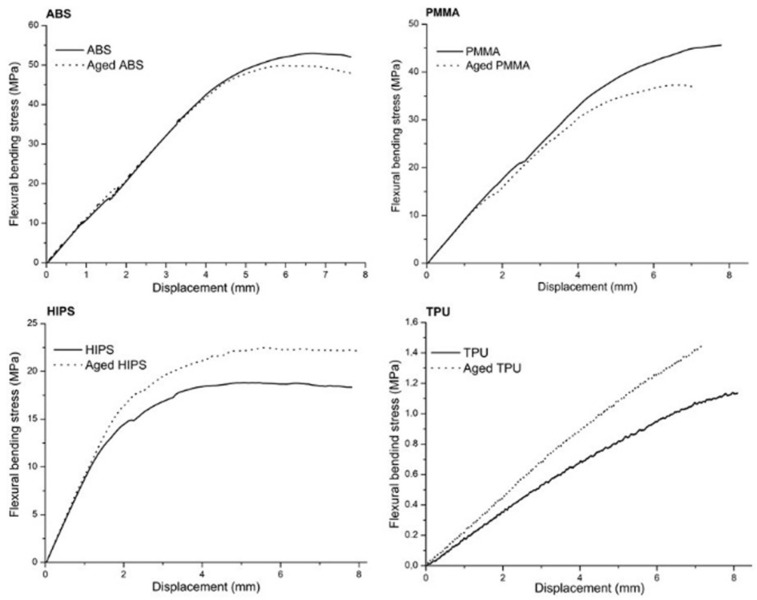
Stress–displacement curves that were obtained from the 3PB tests of the mono-materials.

The 3PB tests assessed the maximum flexural bending stress (σ_max_) and flexural modulus (*E*) of the as-printed and aged mono-material specimens. The resulting bending/displacement plots are shown in Figure 2.

**Figure 3:**
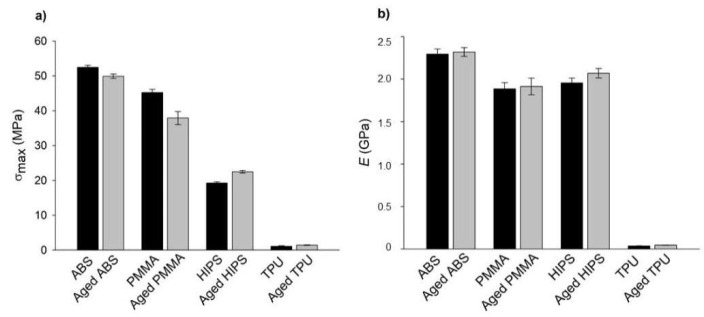
Bar plot representation of (**a**) σ_max_ and (**b**) *E*, which were obtained from the 3PB tests of the mono-materials for the dry and aged specimens.

The plots show that both the dry and aged specimens presented curves with similar trends. However, the flexural bending stress and displacement values were different. The σ_max_ of ABS and PMMA was lower for the aged specimens, while for HIPS and TPU, the opposite was observed (Figure 3).

Although similar studies may be available in the literature, no manuscripts were found that explain the observed trends and values. Most of the published papers state the observed tendencies without fully explaining the obtained results [13,41]. In the present work, and from the observation of Figure 3, it can be stated that the aging process did not significantly influence the mechanical behavior of the tested specimens, except for PMMA. In the previous section, it was observed that the immersion in AS did not significantly alter the dimensions of any printed polymer, which indicated that the solution was not able to penetrate the polymeric networks and cause swelling. Nonetheless, this does not mean that AS did not interact with the polymeric chains, especially those on the outer surfaces. The interaction between the material and saliva solution resulted in a slight increase in the E values for all materials, which means that the water content of the saliva did not act as a plasticizing agent but may indicate that the ions that were present in the solution contributed to increasing the cohesive strength between the macromolecules due to electrostatic affinity [42], increasing the *E* values. 

In what concerns the σ_max_, the aging process did not significantly affect the strength of the studied materials, except, once again, for PMMA. Indeed, for ABS and PMMA, the mean value of σ_max_ decreased with aging, while for HIPS and TPU, this value slightly increased. 

For HIPS and TPU, the increase in cohesive forces due to a possible physical crosslinking that was catalyzed by the ionic strength of the AS could explain the improved resistance to higher loads. However, the same explanation is not valid for PMMA. It is known that the chemical composition and the volume of side groups of polymers influence their mechanical behavior [43,44]. The results for HIPS and TPU are according to previously published work, where aging in artificial saliva also induced better mechanical properties in commercial EVA mouthguards [45]. Furthermore, the retention capacity of commercial EVA mouthguards in the presence of saliva is higher when compared with the dry devices [46].

In the present work, PMMA is the only polymer that does not have aromatic rings in its chemical composition, which may explain the difference in the behavior that was observed for this polymer. Aromatic rings are very large groups that hinder the movement of the polymeric chains, thus limiting the relative movement of the macromolecules during deformation, allowing them to resist higher loads [47]. Under the action of AS, it seems that the absence of aromatic rings contributed to the decrease in the σ_max_ of PMMA. Moreover, the hydrophobic -CH_3_ groups that are present in this polymer’s branched structure may have limited the ions’ access to the macromolecules. Thus, the reinforcement effect that was observed for HIPS and TPU was not achieved in PMMA.

**Figure 4:**
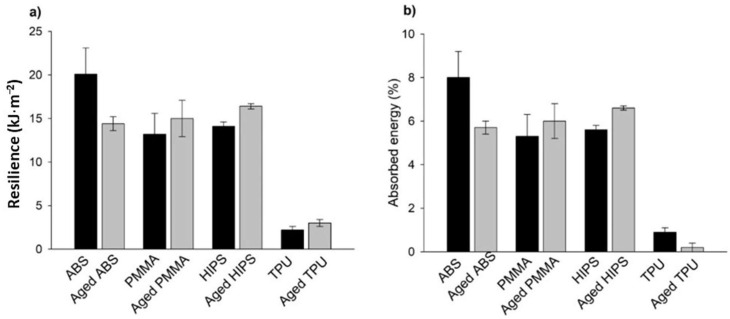
(**a**) Resilience and (**b**) absorbed energy of the printed mono-material specimens, which were obtained using transverse impact tests.

The resilience and absorbed energy of the printed mono-material specimens were obtained through transverse impact tests and the results are presented in Figure 4.

**Figure 5:**
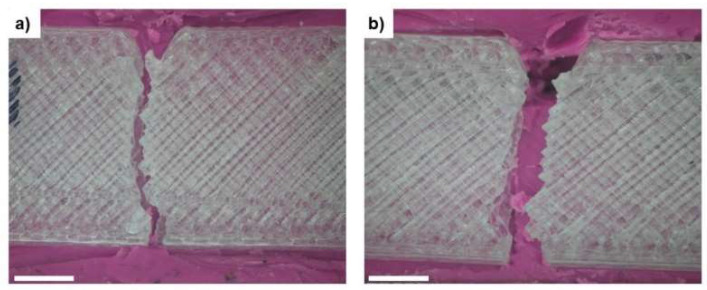
Damage induced by the transverse impact tests on PMMA specimens: (**a**) as printed and (**b**) aged. Scale bar: 500 µm.

Generally, it can be stated that the aging process in AS had no significant influence on the performance of the printed mono-materials under impact tests, except for the ABS specimens. In this polymer, the resilience for aged specimens decreased by 28.7% and the absorbed energy by 34.4% as a consequence of the interactions with the ions in the AS solution. The ionic interactions may be related to the chemical composition of ABS, which was the only polymer with a C≡N bond and an electronegative N present [48]. Both of these factors increase the intermolecular strength of ABS macromolecules [49], which makes intermolecular motion more difficult, thus resulting in less ability to absorb energy and, consequently, producing a lower resilience. Despite the obtained values for all the tested polymers, only PMMA specimens, as printed and aged, suffered from cracking and complete fracture after the impact (Figure 5). 

This observation is a consequence of the fact that, except for TPU, which presents a behavior similar to elastomers, PMMA presents the lowest values of resilience and absorbed energy from all the remaining three polymers. 

The partial conclusion of the characterization made for the printed mono-materials is that, except for the ABS and PMMA, the aging process did not significantly change the mechanical properties of the materials. For PMMA, it seems that the AS solution acted as a plasticizer, decreasing the σ values. However, in this case, the plasticizing effect must have mainly occurred only on the outer shell of the printed specimens since E increased, which is not expected from a plasticized structure. Therefore, such behavior implies that the chemical compounds that were present in the saliva solution could not be absorbed by the polymer and, therefore, did not reach the inside of the printed material. In turn, the aging process on ABS only impaired the resilience and absorbed impact energy, which means that the AS components that were able to interact with ABS improved the ability of the copolymer to dissipate energy.

### 3.3. Mechanical Properties of Printed Sandwich Structures

Through multi-material printing, it is possible to produce parts and devices from materials with different properties, which will result in printed specimens with advanced properties, as described in the literature [13]. In the present work, the combination of materials was achieved by preparing sandwich structures in which TPU was placed as the core in every specimen, as described in the Materials and Methods section. The combination of soft and rigid polymers is more likely to be successful, as pointed out by studies with the bulk EVA copolymer [50].

According to the literature, a 100% linear infill was selected for the skins and core to maximize the adhesion between the different polymers [30]. 

**Figure 6:**
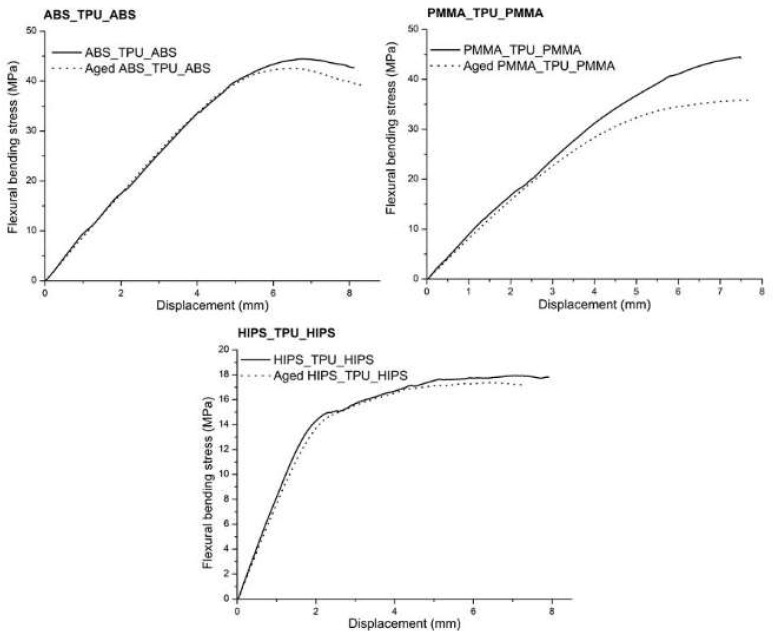
Representative curves of the sandwich structure materials that were obtained using 3PB tests.

The sandwich structures were mechanically tested using the same techniques and conditions as the mono-material. The determination of σ_max_ and *E* was performed using 3PB tests, and the representative flexural bending stress–displacement curves that were obtained are displayed in Figure 6.

**Figure 7:**
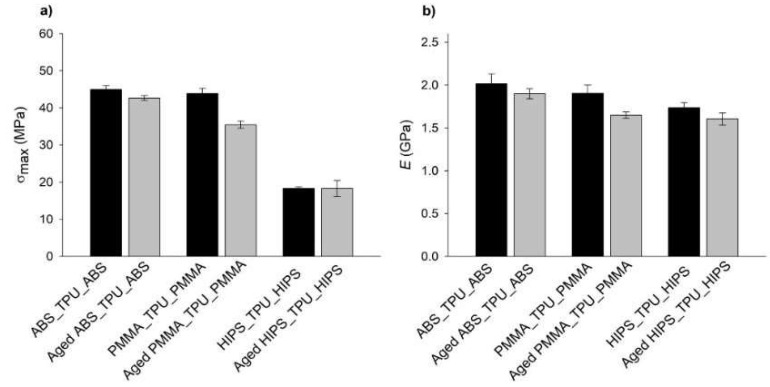
Determined (**a**) σ_max_ and (**b**) *E* values of the dry and aged sandwich structure specimens that were obtained from the 3PB tests.

The behavior of all the sandwich structures was slightly influenced by the aging process, mainly in the plastic deformation regime. Herein, although the plot trends were very similar, the flexural bending stresses of the aged structures were lower than the as-printed samples, especially for the PMMA_TPU_PMMA sandwich. The calculated σ_max_ and *E* values are summarized in Figure 7. 

The inclusion of TPU in the sandwich structures resulted in a decrease in σ and *E* for both the as-printed and aged specimens in all the tested materials, as expected. Considering that one-third of the overall volume of the specimen was composed of TPU, which is a polymer with very low values for the tested mechanical properties, its presence decreased the value of the mechanical properties of the other tested mono-material specimens.

Nevertheless, it must be highlighted that aging tests did not significantly affect the mechanical behavior of the multi-material specimens, in opposition to what happened in the mono-material specimens. The reason appears to be due to the incorporation of TPU, which, as a mono-material, showed a more homogeneous mechanical behavior in the presence of artificial saliva. 

However, for the PMMA-containing structures, the decrease in σ_max_ was more pronounced. Such a fact may be explained by the behavior of PMMA as a single material and the lower adhesion between this polymer and TPU due to the already mentioned difference in the chemical composition. The lower adhesion enabled the artificial saliva to reach deeper into the polymeric network and increase the area of contact with the AS. Once penetrating the polymeric network, the water that was present in the artificial solution may have acted as a plasticizing agent, leading to the decrease in both σ_max_ and *E* [51].

**Figure 8:**
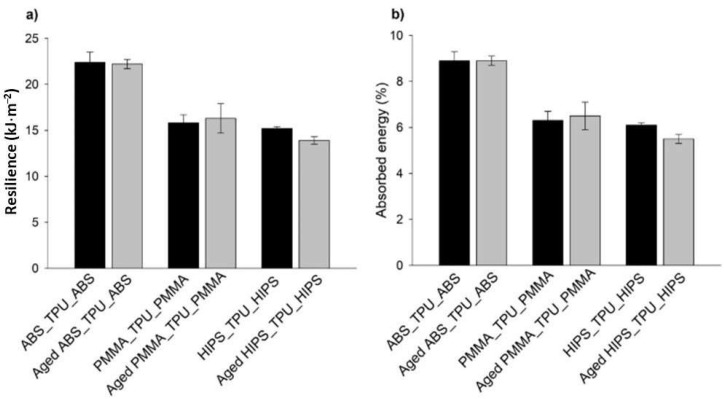
(**a**) Resilience and (**b**) absorbed energy, which were determined by transversal impact tests.

The absorbed energy and resilience of the sandwich structure materials that were determined using transversal impact tests are plotted in Figure 8.

The inclusion of TPU in the multi-material sandwich structures resulted, overall, in stabilizing the values that were observed before and after the aging process for all materials, which is a very significant result, as explained before.

Considering the resilience and absorbed energy standard deviations (maximum and minimal values), it was observed that no significant differences existed between the mono- and multi-materials, with a tendency for a slight increase in the evaluated properties for multi-material specimens.

The only exception that is worth mentioning is the PMMA-TPU-PMMA sandwich structure. In this case, a more significant increase in the mean values was observed. The increase in the resilience and absorbed energy of the tested specimens after the aging process increased by 11% compared to the mono-material results. This observation indicated that the hydration phenomena that occurred due to the lower adhesion between the two different polymers, as already explained, allowed for a more hydrated polymeric network, which induced lower resilience and absorbed energy and, consequently, higher dispersed energy [52].

**Figure 9:**
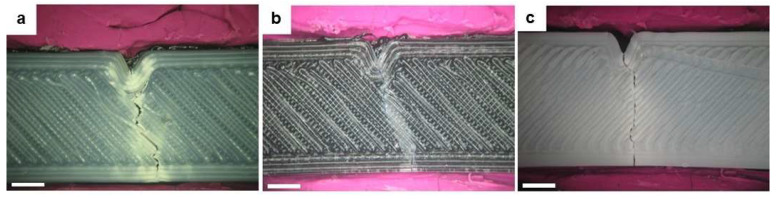
Surface damage of the as-printed sandwich-structured specimens after the transversal impact tests: (**a**) ABS_TPU_ABS, (**b**) PMMA_TPU_PMMA, and (**c**) HIPS_TPU_HIPS. Scale bar: 500 µm.

In what concerns the damage that was induced by the hitting hammer, contrary to what happened for the mono-material structures where only the PMMA completely broke, all sandwich structures showed visible cracking of the skins (Figure 9).

The crack propagation that was observed for ABS, PMMA, and HIPS skins in the sandwich structures may have been related to the thickness of the layers. When tested as a mono-material, the specimens were 2 mm thick, which enabled the structures to resist the impact of the hammer without visible cracking. On the other hand, in the sandwich structures, the thickness of the layer facing the hammer was three times lower than that in the mono-material specimens. Therefore, the decrease in thickness may have been responsible for the fragility of the layer. 

The in-depth propagation of the formed cracks on the sandwich skins stopped when they reached the core TPU. Such behavior was expected since, in the mono-material tests, TPU showed the lowest percentage of absorbed energy, which means that the TPU could successfully dissipate a large ratio of impact energy and maintain its structural integrity without the formation of crack defects. Moreover, the crack nucleation occurred at the vertices of the produced notch, which corresponded to the place with the highest concentration of tension.

It is worth highlighting that the skins that did not face the hammer did not present any visible crack formation, indicating that the developed sandwich structures can be used as oral protective devices, such as mouthguards [53]. Even if the impact suffered by the athlete produces damage in the polymer facing the opponent, the propagation does not travel to the polymer that is in contact with the teeth and gum due to the presence of the TPU core, which dissipates most of the impact energy. Therefore, teeth and maxilla-facial articulations are protected. 

## 4. Conclusions

The present work aimed to evaluate the mechanical properties of three sandwich configurations using different polymeric materials for the skins and core (ABS-TPU-ABS, PMMA-TPU-PMMA, HIPS-TPU-HIPS). Moreover, the influence of the aging process on mechanical performance was studied using artificial saliva. The behavior of the printed multi-material sandwich structures was compared with each of the mono-materials that were printed with the same printing parameters.

From the obtained results, it can be concluded that the presence of saliva had an impact on some of the printed mono-materials. Through the 3PB tests, decreases in the σ_max_ and *E* values were observed after aging for the PMMA specimens, while transverse impact tests revealed that the resilience and the absorbed energy of the ABS specimens were negatively affected by the aging process. These variations could be attributed to the physicochemical interactions between the ionic components of artificial saliva and the chemical groups of the polymeric networks.

In what concerns the sandwich structures, it can be stated that their mechanical performance was not significantly affected by the aging process, probably due to the presence of the TPU used as core material, which acted as a stabilizer. However, combining TPU with other materials negatively affected the bending properties of the printed specimens, as expected, since TPU was the mono-material with the lowest flexural bending stress values. 

The sandwich configurations that presented the best bending properties were the ABS-TPU-ABS and PMMA-TPU-PMMA specimens, as they had the highest values for σ_max_ and similar results for *E*, although the first combination had a higher rigidity before the aging process. It must be highlighted that there were some adhesion problems in the aged HIPS-TPU-HIPS specimens, as the different material layers were separated during the 3PB testing.

Through transverse impact testing, it was possible to conclude that TPU slightly improved the mechanical properties of the printed specimens while decreasing the variability in the results from each test performed. The multi-material with the best results was ABS-TPU-ABS due to the highest resilience value of all material combinations.

## Figures and Tables

**Table 1:** Printing parameters of the printed specimens.

Configuration	Filament	T_printing_ (°C)	T_bed_ (°C)	P_s_ (mm/s)
Mono-Material	ABS	220	110	50
	HIPS	220	110	50
	PMMA	220	110	50
	TPU	220	110	15
Sandwich Structure	ABS-TPU-ABS	220	110	15
	HIPS-TPU-HIPS	220	110	15
	PMMA-TPU-PMMA	220	110	15

T—temperature; Ps—printing speed.

**Table 2:** Mean average and standard deviation values of the dimensions of the printed before (dry) and after (aged) the aging process.

Test	Material	Length (mm)		Width (mm)		Thickness (mm)	
		Dry	Aged	Dry	Aged	Dry	Aged
**3PB**	ABS	59.8 *	59.9 *	10.0 *	10.0 *	2.1 *	2.1 *
	HIPS	59.8 *	59.7 ± 0.1	9.8 ± 0.4	10.0 *	2.1 *	2.1 *
	PMMA	59.9 *	60.2 ± 0.1	10.2 *	10.2 *	2.1 *	2.1 *
	TPU	59.9± 0.2	60.2 ± 0.1	10.4 ± 0.2	10.4 ± 0.2	2.1 ± 0.2	2.1 ± 0.2
	ABS-TPU-ABS	60.3 ± 0.1	60.3 ±0.1	10.4 ± 0.1	10.4 ± 0.1	2.4 ± 0.1	2.4 ± 0.1
	HIPS-TPU-HIPS	60.3 ± 0.1	60.2 ± 0.1	10.4 ± 0.1	10.3 ± 0.1	2.3 *	2.4 *
	PMMA-TPU-PMMA	60.1 ± 0.1	60.3 ± 0.1	10.4 ± 0.1	10.4 ± 0.1	2.5 *	2.5 *
Transverse impact	ABS	79.5 ± 0.1	79.6 ± 0.1	9.9 *	9.9 *	2.1 *	2.1 *
	HIPS	79.6 ± 0.1	79.5 *	9.9 *	9.9 *	2.1 *	2.1 *
	PMMA	79.8 ± 0.1	80.0 ± 0.1	10.1 ± 0.1	10.1 ± 0.1	2.1 *	2.1 *
	TPU	80.2 ± 0.1	80.3 ± 0.1	10.5 ± 0.3	10.6 ± 0.1	2.1 ± 0.1	2.2 ± 0.2
	ABS-TPU-ABS	84.0 ± 0.1	84.1 *	10.7 *	10.8 *	2.4 *	2.4 *
	HIPS-TPU-HIPS	84.0 ± 0.1	84.0 ± 0.1	10.8 *	10.8 *	2.4 *	2.4 *
	PMMA-TPU-PMMA	84.1 ± 0.1	84.3 ± 0.2	10.9 ± 0.1	10.9 ± 0.1	2.5 *	2.5 *

* Values with a standard deviation of 0.0.

## Data Availability

Data available upon request.
